# Interpreting the role of nuchal fold for fetal growth restriction prediction using machine learning

**DOI:** 10.1038/s41598-022-07883-0

**Published:** 2022-03-10

**Authors:** Lung Yun Teng, Citra Nurfarah Zaini Mattar, Arijit Biswas, Wai Lam Hoo, Shier Nee Saw

**Affiliations:** 1 Department of Information Technology, Faculty of Computer Science and Information Technology, Universiti Malaya, 50603 Kuala Lumpur, Malaysia; 2 Department of Obstetrics and Gynecology, Yong Loo Lin School of Medicine, National University of Singapore, Singapore, Singapore; 3 Department of Obstetrics and Gynaecology, National University Health System, Singapore, Singapore; 4 Department of Artificial Intelligence, Faculty of Computer Science and Information Technology, Universiti Malaya, 50603 Kuala Lumpur, Malaysia

**Keywords:** Diagnosis, Intrauterine growth

## Abstract

The objective of the study is to investigate the effect of Nuchal Fold (NF) in predicting Fetal Growth Restriction (FGR) using machine learning (ML), to explain the model's results using model-agnostic interpretable techniques, and to compare the results with clinical guidelines. This study used second-trimester ultrasound biometry and Doppler velocimetry were used to construct six FGR (birthweight < 3rd centile) ML models. Interpretability analysis was conducted using Accumulated Local Effects (ALE) and Shapley Additive Explanations (SHAP). The results were compared with clinical guidelines based on the most optimal model. Support Vector Machine (SVM) exhibited the most consistent performance in FGR prediction. SHAP showed that the top contributors to identify FGR were Abdominal Circumference (AC), NF, Uterine RI (Ut RI), and Uterine PI (Ut PI). ALE showed that the cutoff values of Ut RI, Ut PI, and AC in differentiating FGR from normal were comparable with clinical guidelines (Errors between model and clinical; Ut RI: 15%, Ut PI: 8%, and AC: 11%). The cutoff value for NF to differentiate between healthy and FGR is 5.4 mm, where low NF may indicate FGR. The SVM model is the most stable in FGR prediction. ALE can be a potential tool to identify a cutoff value for novel parameters to differentiate between healthy and FGR.

## Introduction

Fetal Growth Restriction (FGR) is a common obstetrical pathology that can limit a fetus from reaching its biologically determined growth potential. FGR is a leading cause of perinatal morbidity and mortality. The prevalence of FGR is approximately 5–25% in developing countries^1^. FGR fetuses have a higher risk of complications, which include stillbirths, faltering growth, prematurity, and adverse neurodevelopmental outcomes. They are at a higher risk of significant long-term non-communicable diseases, such as diabetes mellitus and cardiovascular conditions^2^.

Various machine learning (ML) models in predicting FGR using different risk factors have been proposed^3–15^. ML models outperform traditional models in predicting FGR using multiple combinations of risk factors such as maternal demographics^7^, medical history, maternal serum biomarkers^8^, and fetal ultrasound measurements^11^. So far, the best ML model achieved an accuracy of 93% in predicting FGR in 34–38th weeks^4^. Despite achieving high prediction accuracy, the deployment of ML models in real clinical settings is rare. In healthcare, clinicians often find it challenging to trust the complex ML model prediction unless we can understand the reasoning of the 'models' prediction.

To develop trust in the complex ML model prediction in the medical field, model-specific and model-agnostic interpretability techniques are introduced. Model-specific techniques are limited to specific models where an explanation is derived from the model. Linear regression and decision trees are examples of model-specific techniques used in healthcare for more than two decades. On the other hand, model-agnostic techniques have been introduced recently^16^. It is mainly divided into global and local interpretation methods. Global and local interpretation methods have been proposed to transform a black-box model into a white-box model. Global interpretable methods are primarily used to understand the effect of features on model prediction based on the entire dataset. In contrast, local interpretable methods help understand a single prediction when input is perturbed. A detailed interpretable ML model in healthcare applications can be found in Stiglic et al.^17^.

Our previous study revealed that the nuchal fold (NF) was an essential parameter in predicting FGR at birth^11^. This study aims to deepen the previous analysis work^11^ using data inherited from previous work to provide an explanation of the model’s prediction using both global and local interpretability techniques. Such techniques can be useful for clinicians to understand the reasoning behind the model’s prediction. In this study, we carried out analysis by (i) investigating the effect of NF in predicting FGR using six ML models, (ii) explaining the ML results using model-agnostic interpretable techniques, and (iii) comparing the 'threshold' obtained from the ML model to differentiate between normal and FGR with that of in clinical guidelines. Our study found that low NF may indicate FGR.

## Results

The code used in this study is available at https://github.com/leonardteng/FGR_XAI.

### Feature correlation

**Figure 1:**
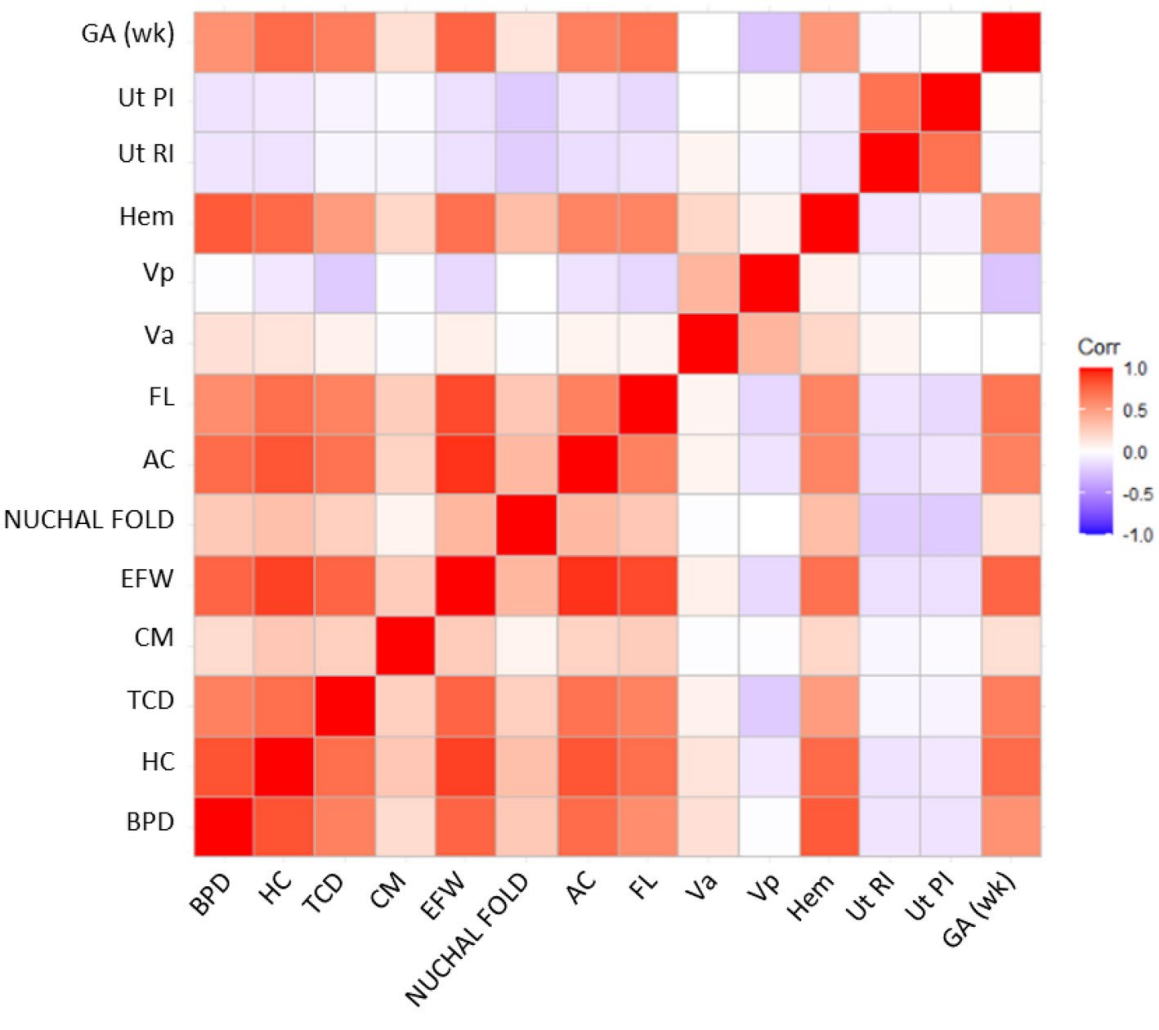
Correlation plot. Uterine artery (Ut) PI and RI show a relatively weak correlation with other antenatal ultrasound measurement variables within ± 0.25. Nuchal fold also has a weak correlation with all other variables, which is within ± 0.37. *BPD* biparietal diameter, *HC* head circumference, *TCD* transverse cerebellar diameter, *CM* cisterna magna, *EFW* estimated fetal weight, *AC* abdominal circumference, *FL* femur length, *Va* anterior horn of the lateral ventricle, *Vp* posterior horn of the lateral ventricle, *Ut RI* uterine resistance index, *Ut PI* uterine pulsatility index, *GA* (*wk*) gestational age measured in weeks.

A correlation plot (Fig. 1) illustrates that Uterine Doppler indices have a weak correlation with other parameters, where all correlations are below 0.25. NF had a low correlation (below 0.37) with all variables. On the other hand, fetal biometry measurements such as Biparietal diameter (BPD), Head circumference (HC), Transverse cerebellar diameter (TCD), Estimated fetal weight (EFW), Abdominal circumference (AC), Femur length (FL), and fetal cerebral Hemisphere (Hem) had high correlations with each other. One interesting point that we observed was that central nervous system (CNS) related features—the Cisterna Magna (CM) and the Anterior Horn of Lateral Ventricle (V_a_), the Posterior Horn of Lateral Ventricle (V_p_) hardly correlate with each other.

### Feature importance analysis

**Figure 2:**
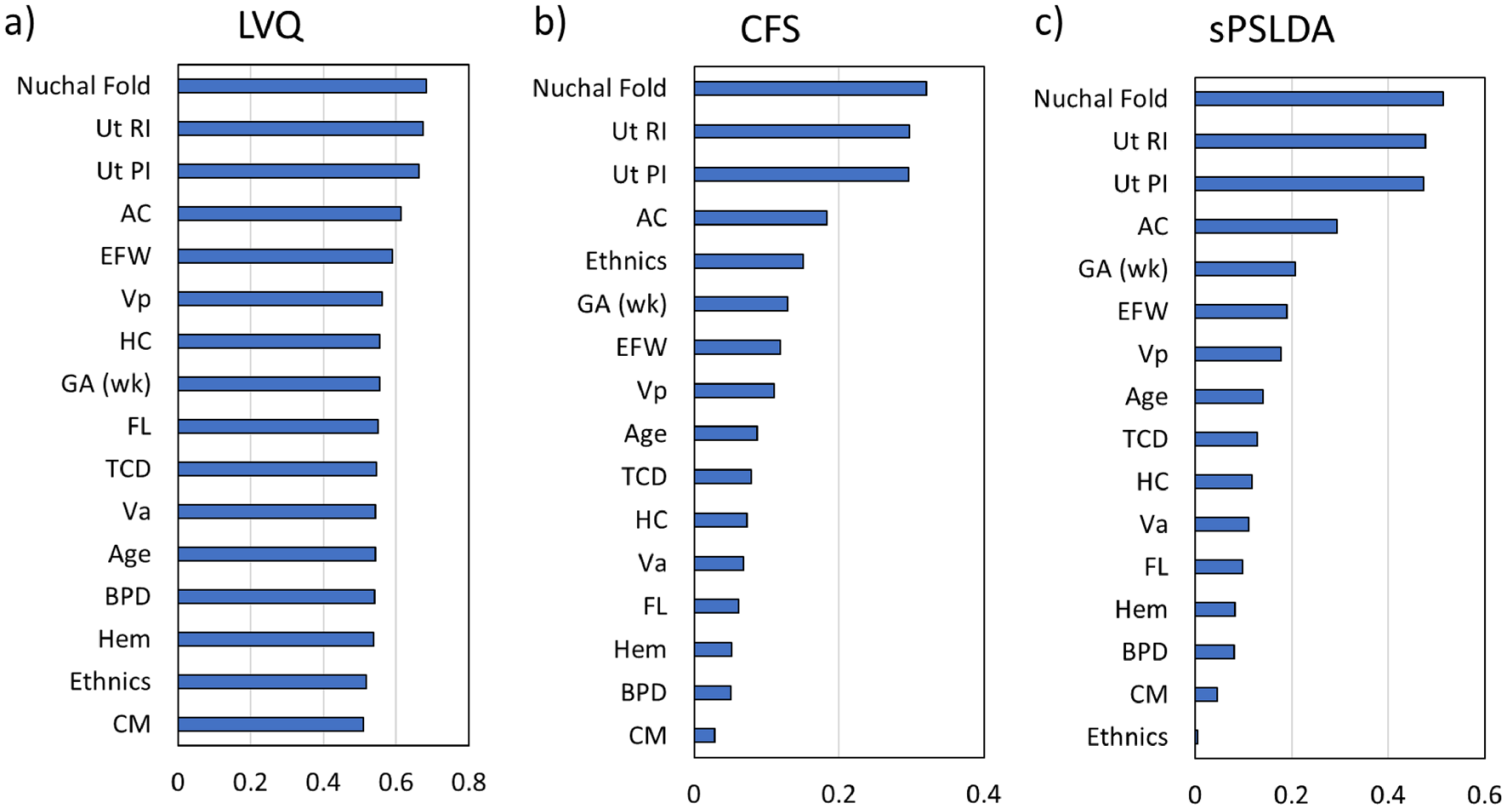
Predictor variable features sorted in descending order of importance using different feature importance methods—(i) Learning Vector Quantization (LVQ), (ii) Correlation-based Feature Selection (CFS), and (iii) Sparse Partial Least-Squares Discriminant Analysis (sPLSDA). NF, Ut RI, Ut PI, and AC appear to be the top four important predictors shown by all methods. *BPD* biparietal diameter, *HC* head circumference, *TCD* transverse cerebellar diameter, *CM* cisterna magna, *EFW* estimated fetal weight, *AC* abdominal circumference, *FL* femur length, *Va* anterior horn of the lateral ventricle, *Vp* posterior horn of the lateral ventricle, *Ut RI* uterine resistance index, *Ut PI* uterine pulsatility index, *GA* (*wk*) gestational age measured in weeks.

Figure 2 represents the features sorted in descending order of importance for each feature selection method used—Correlation Based Feature (CFS), Sparse Partial Least-Squares Discriminant Analysis (sPLS-DA), and Learning Vector Quantization (LVQ). All methods consistently showed that NF was the best predictor for FGR prediction, followed by Ut RI, Ut PI, and AC. The feature importance value for NF, Ut RI, and Ut PI was consistently higher than the other features.

### Effect of nuchal fold on machine learning models performance

**Figure 3:**
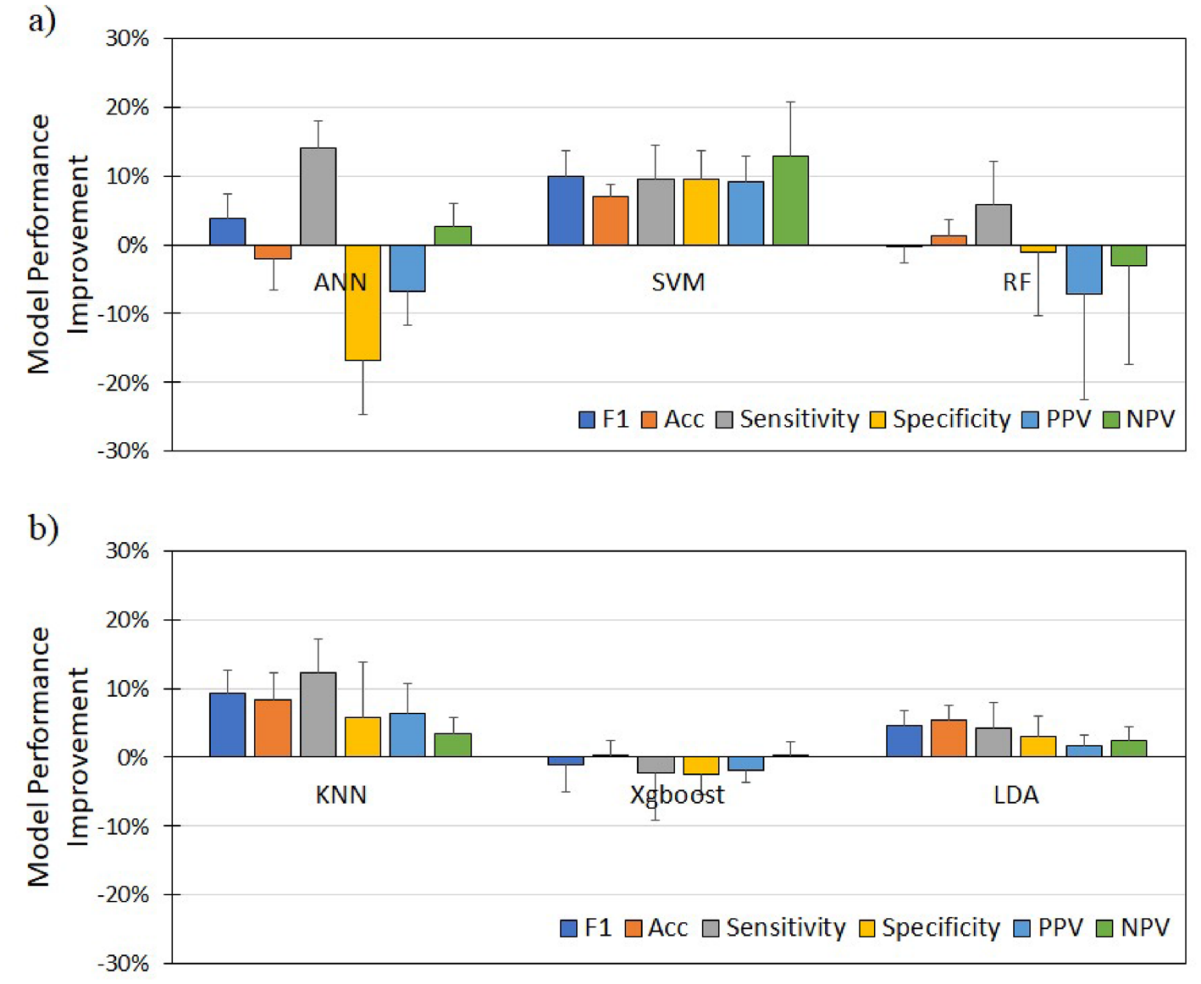
Discriminative performance improvement between models with and without Nuchal Fold. The value shown is the mean and standard error of 10 cross-validation experiments for Artificial Neural Network (ANN), Support Vector Machine (SVM), and Random Forest (RF). *PPV* positive predictive value, *NPV* negative predictive value.

The feature importance analysis found that NF was the top contributor in predicting FGR. We, thus, compared the ML models' performance with and without NF. Figure 3 shows the performance metrics improvement, using six ML models: (i) Artificial Neural Network (ANN), (ii) Support Vector Machine (SVM), (iii) Random Forest (RF), (iv) K-Nearest Neighbor (KNN), (v) XgBoost, and (vi) Linear Discriminant Analysis (LDA) models, after the addition of NF. The SVM, KNN, and LDA models show consistent improvement after adding NF into the model for all evaluation metrics, which are F1, accuracy, sensitivity, specificity, Positive Predictive Value (PPV), and Negative Predictive Value (NPV). Among these models, the SVM model had the highest consistent improvement across all performance metrics in classifying healthy and FGR. The ANN model had fluctuating performance, showing improvement in F1, sensitivity, and NPV but otherwise for the accuracy, specificity, and PPV. RF and XgBoost models only show improvement in sensitivity and accuracy, respectively, after adding NF but decrement in other performance metrics. The SVM model was considered the best model, delivering consistent results and the most remarkable improvements in all evaluation metrics among all the models. Thus, the SVM model was chosen for interpretability analysis.

### Interpretable analysis

The most important feature—NF, as indicated in Fig. 2, was checked for its interaction strength with the other ultrasound measurement data. Table 1 shows the top five interaction strengths of NF with other variables. The variables with the highest interactive strength with NF were Gestational Age (GA) at the time of scanning, AC, FL, ethnicity, and maternal age. The variable with the highest interactive strength with NF was only 0.387, which was weak, suggesting that NF, albeit the most important feature, its predictive strength was hardly influenced by any other parameters.

**Table 1:** Top five interactive strengths of the Nuchal Fold with other antenatal ultrasound measurements data in Support Vector Machine.

Support vector machine		Interactive strength
Nuchal fold	GA at the time of scanning	0.387
	Abdominal Circumference	0.353
	Femur length	0.331
	Ethnicity	0.325
	Maternal age	0.254

**Figure 4:**
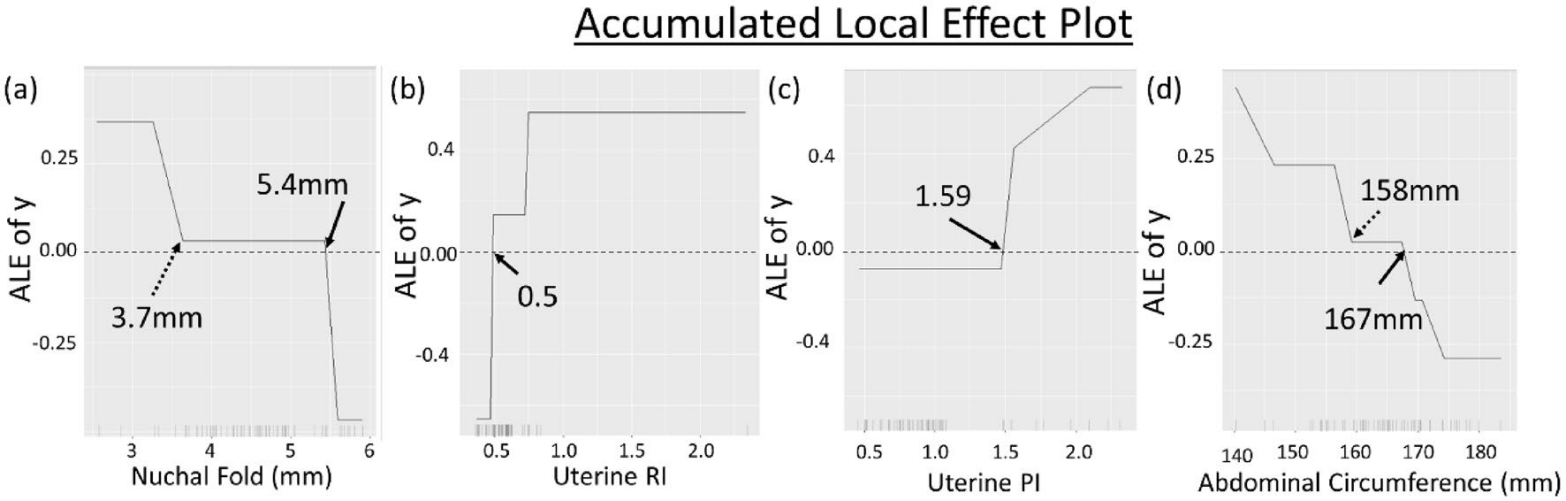
Accumulated local effect (ALE) plot of the top four features—(**a**) Nuchal Fold, (**b**) Uterine RI, (**c**) Uterine PI, and (**d**) Abdominal Circumference from the Support Vector Machine (SVM) in predicting FGR. Nuchal fold below 3.7 mm, Ut RI above 0.5, Ut PI above 1.59, and AC below 158 mm showed an increased probability of fetuses being born as FGR.

Figure 4 shows the Accumulated Local Effect (ALE) plots for the top four features—(a) NF, (b) Ut RI, (c) Ut PI, and (d) AC. The four ALE plots explain the effect of these features in affecting the FGR prediction from the SVM model. The plots are centered at zero, which indicates the average model prediction across all variable values. Positive ALE values indicate a higher chance of FGR, while negative ALE values indicate a lower chance of FGR (high chance of healthy). In Fig. 4a, the thinner the NF, the higher the chance of fetuses classified as FGR. The probability of the fetus being predicted as an FGR significantly increased when NF was below 3.7 mm, judging from the sharp upward slope. After that, ALE of NF has a constant value and crosses zero at 5.4 mm, indicating this 5.4 mm is a cutoff point to differentiate between healthy and FGR. Figure 4b,c show a substantial increase of FGR prediction when Ut RI and Ut PI cross 0.50 and 1.59, respectively. Moreover, we observed that the cutoff value for AC to differentiate between healthy and FGR is 167 mm, and AC below 158 mm increased the chances of the babies being born as FGR. Our results were comparable with the clinical guidelines where the critical value of Ut RI, Ut PI, and AC to identify the fetus at risk of FGR were 0.59^18^, 1.47^19^, and 150 mm^20^, respectively. The errors between the critical value obtained from our model and the clinical guidelines were 15%, 8%, and 11% for Ut RI, Ut PI, and AC, respectively.

**Figure 5:**
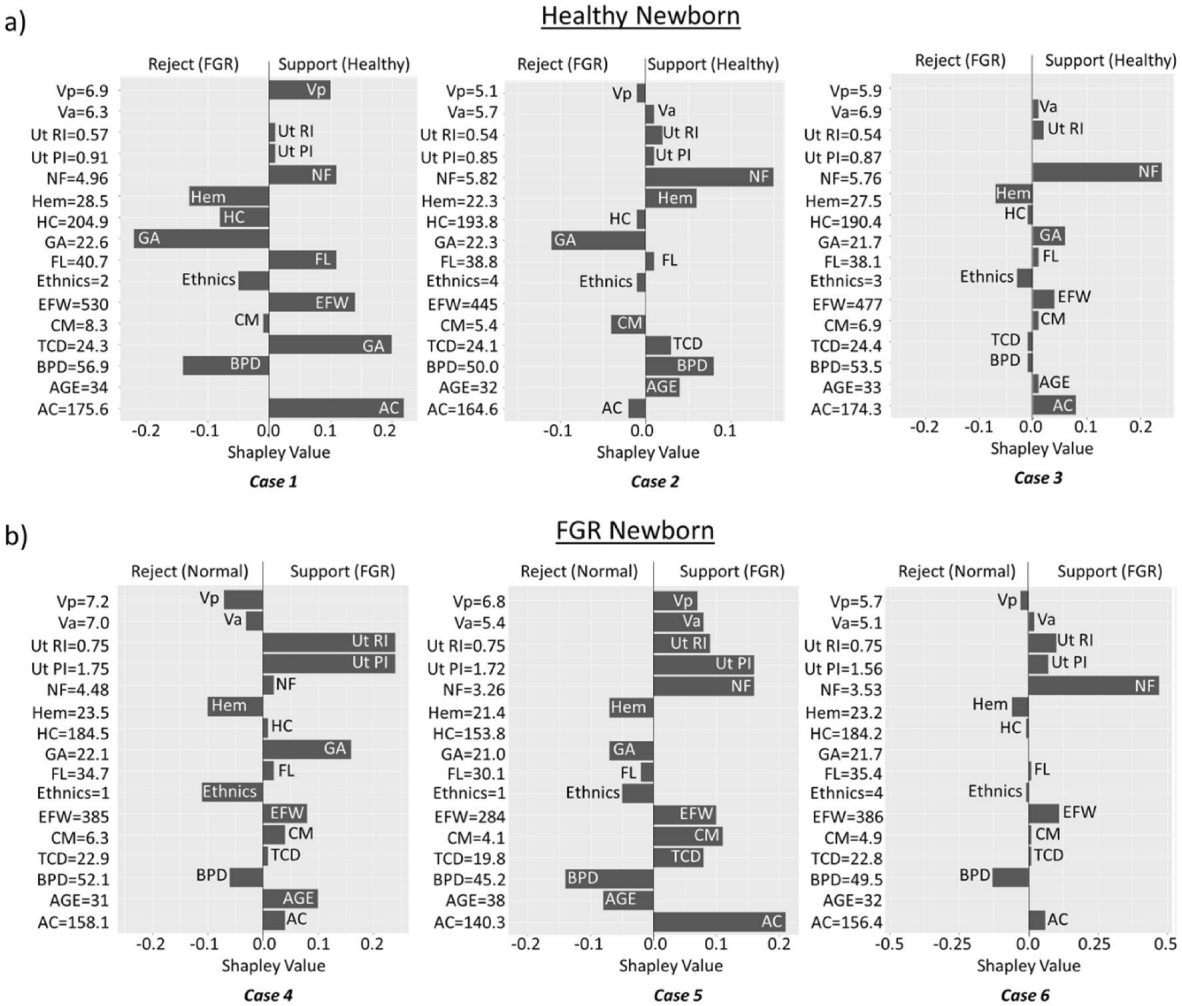
SHAP results for correctly predicted cases of (**a**) normal and (**b**) FGR. The y-axis shows all the features and their respective values for that particular case. The x-axis shows the Shapley value, positive and negative values indicate that feature supports and rejects healthy prediction.

Figure 5 shows the SHAP (Shapley Additive Explanations) summary plots, which illustrate the feature contributions to the model prediction for any particular case; it provides positive (support) and negative (reject) relations of each feature to the prediction^21,22^. Figure 5 illustrates six SHAP summary plots from six representative cases predicted correctly by the SVM model. SHAP summary plots allow us to understand the reasoning of the ML model prediction. For example, the model correctly predicted case 1 as a healthy baby. We observed that the variables that supported the outcomes were Vp, Ut RI, Ut PI, NF, FL, EFW, GA, and AC. In Case 1, AC had the largest Shapley value, suggesting that AC played the largest role in affecting the model's prediction. From the plot, we can see that case 1 fetus had a large AC, 175.6 mm, which far exceeded the critical value (158 mm) in differentiating between healthy and FGR, which explained why Case 1 was predicted by healthy. As for Case 2 and Case 3, the largest Shapley value variable was NF. Case 2 and 3 had large NF (> 5.4 mm), which was why they were predicted to be healthy.

Figure 5b shows the SHAP summary plots for FGR cases. We observed that Ut RI, Ut PI, NF, and AC features that contribute the most. Interestingly, in Case 6, although the Ut PI was large, 1.56 (above the 95th centile^18^), it was not the primary contributor to FGR prediction. The main contributor variable that led to FGR prediction was NF, suggesting that thin NF may aid in predicting FGR and thus require clinical attention during FGR prediction.

## Discussion

In the past years, a few studies deployed ML for FGR detection using antenatal information^23–25^. However, all these studies focus on the model's prediction performance. This paper formulated a pipeline of data analytics techniques to interpret the model's results and compared them with the clinical guidelines.

This study pioneers in reporting the estimation of NF thickness in the second trimester to differentiate FGR from healthy newborns to the best of the authors' knowledge. NF measurements in the second trimester are often used to detect Down Syndromes^26–28^, and thus there is a lack of study investigating the relationship between FGR and NF. Our results (Fig. 4a) show that NF reduction increases fetuses' chance of being born with FGR. The probability of fetuses being born with FGR sharply increases when NF falls below 3.7 mm.

Three feature selection methods—LVQ, CFS, and sPLSDA consistently showed that Ut RI and Ut PI were the second and third top features, followed by AC in predicting FGR. SHAP analysis results were in agreement with the feature selection results. These results are not surprising because abnormal Ut RI and Ut PI had shown to affect placental vasculature developments and thus affect fetus growth^29^. Reduced AC is often observed in FGR due to the brain-sparing effect, in which nutrients are supplied to the brain in a state of hypoxia^30^. One important finding is that all three feature selection methods consistently show that NF is the most important feature in FGR prediction. Furthermore, the SHAP summary plots (Fig. 5) indicate that NF has a high contribution to the model prediction for most cases, where FGR fetuses had thinner NF than healthy fetuses. This finding is in agreement with Saw et al., who reported that NF had the highest importance in predicting FGR using the Random Forest model^11^.

From Fig. 1, the correlation between Ut RI and Ut PI is high, approximately 0.8. Ut PI and Ut RI are highly correlated. Although Ut RI and Ut PI have a high correlation, the interaction effect is moderately low (interaction effect = 0.340 using 2-way H-statistic). The low interaction effect suggests that the interaction effect of Ut RI and Ut PI does not affect much on FGR predictive strength. A previous study corroborated our findings. They reviewed the role of Uterine Doppler (Ut RI, PI, notching) found that increased PI alone and increased PI with notching are both associated with an increased incidence of adverse pregnancy outcomes^31^.

ALE technique has been used in other domains to interpret the effect of predictors variables in the "black-box" supervised model^32–35^. From the ALE plots, we found that the threshold values of Ut RI, Ut PI, and AC to differentiate FGR from healthy fetuses are comparable with the clinical guidelines. For example, the threshold values obtained from ALE plots (Ut RI: 0.50; Ut PI: 1.59) and the clinical guidelines (Ut RI: 0.59, Ut PI: 1.47) differed by approximately 15% and 8%, respectively. A similar finding was observed for AC in which the threshold value from ALE for AC was 11% lower than that suggested in the clinical guidelines (ALE plots: 167 mm vs. Clinical Guidelines: 150 mm). These findings indicate that ALE plots could be useful in defining the cutoff value of certain features in differentiating between healthy and FGR, especially on the feature that yet has a clinical cutoff guideline, such as the NF in this study.

Other important issues that are often neglected during data exploration are feature interaction and feature redundancy. When there is high interaction between two features, putting them together into the model may change the classification result completely. A completely non-relevant feature may drastically improve the classification performance with a high interactive strength feature. We observed that there was hardly any interaction between NF and other predictors. The most increased feature interaction was with Gestational Age at the time of scanning with a feature interaction of 0.387, suggesting that 'other predictors hardly influenced its' predictive strength. Thus, NF can be said to be serving as a complementary feature for FGR prediction.

It is crucial to have a stable model whose performance should be low variance when deployed in real clinical settings. The SVM model is the most suitable because it had the most consistent results across all performance metrics. Thus, it is suitable to be used as an objective evaluation tool for assessing fetal growth.

There are limitations to the study. Firstly, the sample size in our study is small. Secondly, we only used birth weight to define FGR. Other predictors such as ‘newborn's head circumference’, ‘newborn's length’, and maternal pregnancy information^36^ were omitted. The reason is that our data were collected retrospectively, and some of the information was not available. Thirdly, our definition of FGR was based on birthweight below the 3rd centile^36^, which was a population with more severe growth restriction. Thus, this trade-off would fail to identify neonates with FGR in the 3rd to 10th percentile range. In the future, it would be better if all these biomarkers defined in the Beune et al. study's consensus^36^ could be included to refine the FGR labels. Forth, according to a multicenter study in Thailand^37^, the highest intra- and inter-observer variability for NF were 0.33 mm and 0.40 mm, respectively. In view of fetuses with chromosomal abnormalities, normally with NF > 6 mm, further study needs to be carried out to evaluate the effectiveness of NF cutoff value of 5.4 mm in predicting FGR considering the intra- and inter-observer variability.

To conclude, we demonstrated that (i) ALE plots could be useful in identifying a cutoff value to differentiate between control and FGR, and (ii) SHAP analysis could be useful in providing the reasoning of the model's prediction. Our data analytic pipeline to interpret the model's results and the effect of features can be easily extended to other disease predictions to visualize the effects of predictor variables in 'black-box' supervised machine learning models. Moreover, our pipeline can also allow additional features such as maternal medical and obstetrics history to be included and analyze their effects on the prediction.

## Methods

### Overview

Feature importance using CFS, sPLSDA, and LVQ were performed to gauge the importance of all features in FGR predictions. Our previous study found that NF was the most important feature in predicting SGA at birth^11^. We extend our analysis of the contribution of NF in this study. In many classification problems, a feature that is impotent by itself sometimes can provide a significant performance improvement when taken with others, which is why feature interaction is taken into consideration to analyze the addition of NF, which is done using a 2-way H-statistics. We also utilized ALE to interpret NF's role in FGR prediction globally. Next, we used SHAP to explain prediction cases. In this study, R version 4.0.2 and WEKA 3.8.4 (https://waikato.github.io/weka-wiki/downloading_weka/#windows) were used.

### Ethical statement

The waiver of informed consent and study protocol were approved by the National Healthcare Group (Singapore) Domain Specific Review Board (DSRB no:2014/01267). The methods conducted were done in accordance with local regulations.

### Data collection

This is a retrospective study of 242 women with singleton pregnancies who had routine antenatal care and delivered at the National University Hospital Singapore (NUHS). The birth weight percentile was computed based on the INTERGROWTH-21st chart^38^, considering the gestational age and newborn gender. Cases, where fetuses exhibited cardiovascular, structural, or chromosomal abnormalities, were excluded from the analysis. Based on the Beune consensus^36^, we defined our FGR classes as newborns with a birth weight lower than the 3rd centile while the healthy with a birth weight higher than the 10th centile^36^. Pregnancies were dated by last menstrual period and confirmed by first‐trimester ultrasound measurements of fetal crump‐rump length^39^. In total, we had a total of 145 healthy and 97 FGR cases. There is no perinatal or maternal death in our population. The average 1 min and 5 min APGAR scores were 8.9 ± 0.7 and 9.0 ± 0.1. Patient characteristics is available in Table 1 (healthy—BW > 10th centile and FGR—BW < 3rd centile) from our previous work^11^.

Table 2 below summarizes the data we obtained for this study. There are a total of 16 features collected in the second trimester, such as fetus gestational age at the time of scanning, Maternal Age, Ethnicity, BPD, TCD, HC, FL, NF, AC, CM, Hem, Va, Vp, EFW, Ut RI and Ut PI. Fetal biometry and Doppler measurements were measured by trained sonographers following ISUOG guidelines^39,40^. NF was measured according to the protocol described in Kim et al. study, in which NF was measured on the transcerebellar plane of the fetal head using calipers placing on the outer edge of the echogenic line of the occipital bone^41^.

**Table 2:** Data collected during the second trimester as predictor and target variables during machine learning model development.

Variable	Features	
Predictor variables	Common features	(i) Fetus gestational age at the time of scanning, measured in weeks (ii) Maternal age (iii) Ethnicity
	Antenatal ultrasound measurements features	(i) Biparietal diameter (ii) Transverse cerebellar diameter (iii) Head circumference (iv) Femur length (v) Nuchal fold thickness (vi) Abdominal circumference (vii) Cisterns magna (viii) Fetal cerebral hemisphere (ix) Anterior horn of the lateral ventricle (x) Posterior horn of the lateral ventricle (xi) Estimated fetal weight
	Doppler features	(i) Uterine Artery Resistive Index (ii) Uterine Artery Pulsatility Index
Target variables		(i) Birthweight centile, computed from INTERGROWTH-21st chart^38^

### Feature correlation

We first performed a multicollinearity test on the data. We used the function of *ggcorrplot* in R to construct a correlation plot to visualize the collinearity among the predictor variables. The correlation matrix is computed based on Eq. (1) on all the predictor variables.

1 $$r_{jk} = \frac{{S_{jk} }}{{S_{j} S_{k} }} = \frac{{\mathop \sum \nolimits_{i = 1}^{n} \left( {x_{ij} - \overline{{x_{j} }} } \right)\left( {x_{ij} - \overline{{x_{k} }} } \right)}}{{\sqrt {\mathop \sum \nolimits_{i = 1}^{n} \left( {x_{ij} - \overline{{x_{j} }} } \right)^{2} } \sqrt {\mathop \sum \nolimits_{i = 1}^{n} \left( {x_{ij} - \overline{{x_{k} }} } \right)^{2} } }}$$

where, $r_{jk}$ = the correlation between predictor *j* and *k, *$S$ = variance, $S_{jk}$ = the covariance between predictor *j* and *k, *$x$ = the predictor variable values, $\overline{x}$ = the mean of the predictor variable.

### Feature importance

We implemented three different algorithms to investigate the feature importance in predicting FGR – (i) CFS, (ii) sPLSDA, and (iii) LVQ.

CFS measures a linear correlation between two variables, in which the resulting values are within [− 1, 1]. The negative sign refers to a negative relation and vice versa. This feature selection technique estimates the relationship between the predictor and the target variables. It assumes that features with a low correlation with the target variable are irrelevant. CFS algorithm is implemented in the “Feature Selection” feature of *WEKA* software^42^. PLS-DA is a multivariate dimensionality-reduction tool. It is considered as a “supervised” version of Principal Component Analysis (PCA) since PLS-DA is made aware of class labels in its input. PLS-DA is a discrimination form method based on PLS for classification purposes. PLS-DA explains maximum separation between the defined class of samples. A PLS regression performs PLS-DA against a dummy matrix *Y* that indicates classification group^43^. The statistical information acquired from this PLS-DA model can be used to determine which predictor variables are important in classifying the output classification group, *Y* (target variable)^44^. While the *plsda* function in R software can only be used for classification, the *splsda* function, which performs sparse PLS in R software, embeds feature selection. In R software, these functions are found in the *mixOmics* package. Lastly, LVQ is an exceptional artificial neural network, a competitive network that uses supervised learning. The variable importance produced by the LVQ trained model was generated using the *varImp* function in R software.

### Effect of nuchal fold on machine learning models performance

The feature importance analysis found that NF has the highest importance in predicting FGR. The effect of NF in machine learning models was investigated using six machine learning models—(i) RF, (ii) ANN, (iii) SVM, (iv) XgBoost (v) LDA (vi) KNN. The model performance between the models with NF and without NF was compared.

As there were different scales due to the nature of the respective features, the predictor ‘variables’ features were normalized to z-score to ensure all measurements were in standard scale. The data were randomly split into 10-folds, and a 10-fold Cross-Validation (CV) study was performed. In each fold, the training data was oversampled using the *SMOTE* algorithm. *SMOTE* stands for Synthetic Minority Oversampling Technique, which uses the k-nearest neighbor algorithm to create synthetic data. It is necessary to balance the classes because unequal sampling of the dependent variables can substantially lower the performance of machine learning models^45^. Oversampling-induced balanced datasets generally outperform the imbalanced dataset.

The machine learning 'models' discriminative performance was evaluated using five evaluation metrics—(i) F1 Score, (ii) Sensitivity, (iii) Specificity, (iv) PPV, and (v) NPV. SVM was chosen as the supervised learning model for interpretability analysis because SVM was the most stable model, judging from the consistent improvement in all the evaluation metrics (see Fig. 3).

### Interpretability analysis

The *Interpretable Machine Learning* (*IML*) package in R provides the tools for examining any black-box machine learning models. To illustrate the interpretability of the machine learning model, we used independent test data. Hence, we split our data into two subsets: 75% training data and 25% test data. In the training dataset, we used tenfold cross-validation to tune the model for the best hyperparameters of the SVM model. Then, we created a predictor object, which held the trained model, the test data, and the class labels applied to downstream functions, such as 2-way interaction, ALE, and SHAP functions.

The 2-way interactions between the NF and the other features were performed by using the predictor object. The 2-way interactions examine NF's dependency, which may give rise to the "interaction" that affects the machine learning performance. The interactive strength was investigated using 2-way H-statistics. The equation of the H-statistic is shown in Eq. (2). The H-statistic measures how much of the variation of the predicted outcome depends on the interaction of features. The H-statistic value reflects the interactive strength between variables, and the value is between 0 and 1. An interaction statistic of 0 means that there is no interaction at all. An interaction statistic of 1 between two features means the prediction effect only comes through the interaction between the two features^46^.

2 $$H_{jk}^{2} = \frac{{\mathop \sum \nolimits_{i = 1}^{n} \left[ {PD_{jk} \left( {x_{j}^{\left( i \right)} ,x_{k}^{\left( i \right)} } \right) - PD_{j} \left( {x_{j}^{\left( i \right)} } \right) - PD_{k} \left( {x_{k}^{\left( i \right)} } \right)} \right]^{2} }}{{\mathop \sum \nolimits_{i = 1}^{n} PD_{jk}^{2} \left( {x_{j}^{\left( i \right)} ,x_{k}^{\left( i \right)} } \right)}}$$

where, $PD_{jk} ( {x_{j} , x_{k} } )$ is the 2-way partial dependence of two features, $D_{j} ( {x_{j} } )$ is the partial dependence of a single feature, $PD_{k} ( {x_{k} } )$ is the partial dependence of another single feature.

We performed ALE analysis to visualize the effect of predictor variables with the machine learning 'models' predictions. The principle of ALE is similar to the Partial Dependence Plot (PDP), which describes the effect of features on the machine learning model prediction on average. ALE is faster and unbiased compared to PDP because ALE computes the local effect of one feature towards outcome and works on correlated features^47^. Besides that, ALE plots have more computational advantages compared to PDP plots^47^.

To identify the feature importance, we adopted the SHAP algorithm to the SVM model. SHAP idea originated from game theory, where a prediction can be explained by assuming that each feature value of the instance is a “player” in a game that contributes to the “payout”, which is the prediction. Each feature value of an instance works together to cause a change in the model’s prediction. This total change in prediction among the features is divided to be “fair” to their contributions. SHAP estimates the contribution of each feature to the overall model prediction^21^.
